# Artificial intelligence in pediatrics: promise, peril, and the path ahead

**DOI:** 10.3389/fped.2025.1631521

**Published:** 2025-06-30

**Authors:** Stefan Kurath-Koller

**Affiliations:** Division of Pediatric Cardiology, Department of Pediatrics, Medical University of Graz, Graz, Austria

**Keywords:** artificial intelligence, digital twin, pediatric cardiology, data privacy, ethics, machine learning, healthcare regulation

## 1 Introduction

**Figure 1:**
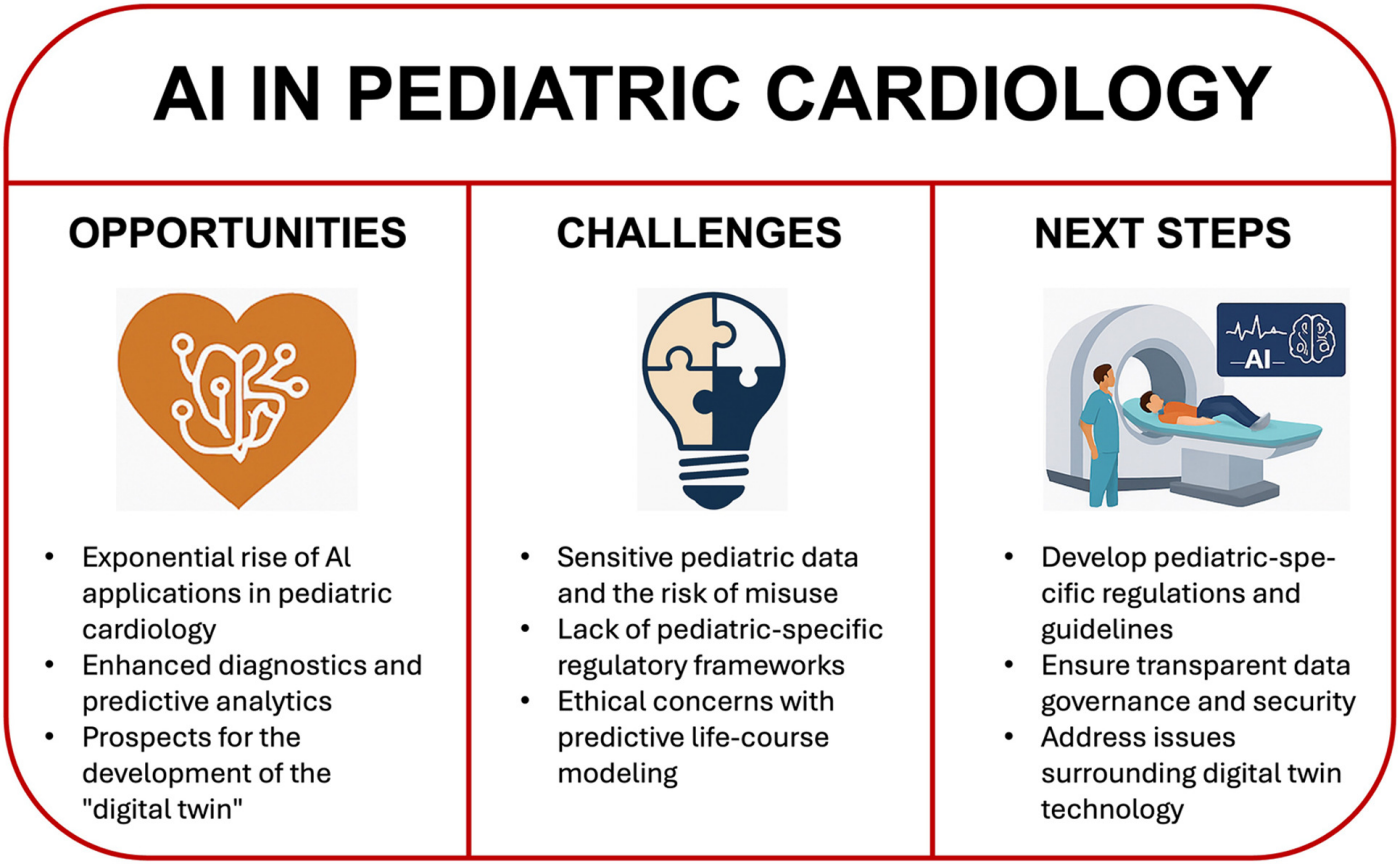
An overview on opportunities, challenges and next steps ahead with regard to AI in pediatric cardiology.

Artificial intelligence has begun to reshape the contours of modern medicine. In pediatrics, this transformation is still in its infancy but is gaining momentum with remarkable speed. Pediatric cardiology, as a data-rich and technology-forward subspecialty, stands at the forefront of this evolution. Yet, unique physiological, ethical, and societal factors make the pediatric application of AI particularly complex—and under-examined. This commentary aims to move beyond an overview and advocate for a dedicated pediatric AI ethics and regulation framework (see Figure 1 for Graphical Abstract).

### 1.1 The rise of AI in pediatric cardiology

1. Emerging Applications AI is being increasingly integrated into clinical workflows in pediatric cardiology. Tools have been developed for automated ECG interpretation, detection of inherited arrhythmia syndromes, segmentation of echocardiographic images, and outcome prediction in congenital heart disease. One recent example is the application of deep learning models to pediatric Apple Watch tracings, which showed diagnostic performance on par with human experts (1, 2).
2. Data-Driven Advances High-dimensional datasets, including genomic panels, wearable sensor data, and multi-frame imaging, can now be processed using machine learning to uncover patterns previously invisible to human interpretation. In pediatric echocardiography, explainable AI models have recently demonstrated clinical utility in real-time image classification, even in neonates and infants (3).
3. From Assistance to Autonomy Traditionally used as diagnostic aids, some AI tools are now exceeding human performance. In a groundbreaking study, AI outperformed clinicians in classifying ECG arrhythmias and predicting one-year mortality—even from normal-appearing tracings (4, 5). This trajectory raises fundamental questions about the evolving role of clinicians in pediatric care.

### 1.2 The digital twin and pediatric-specific ethical dilemmas

1. Digital Twin: Promise and Pressure The concept of the digital twin—a virtual model integrating real-time physiological, behavioral, and genetic data—offers immense potential. In children with congenital or acquired heart disease, it could enable simulation-based personalized therapies, continuous risk monitoring, and adaptive treatment planning (6).
2. Pediatric-Specific Risks Unlike adults, children's developmental trajectories, dependency relationships, and legal consent structures make digital twin deplopment ethically fraught. Predictive modeling across decades could lead to anticipatory discrimination in education or insurance, amplify parental anxiety, and reduce the child's future autonomy. Such risks demand age-specific safeguards.

### 1.3 The regulatory and ethical frontier

1. Pediatric Data Sensitivity—Children's data are vulnerable not only because of identifiability but due to the lifelong implications of predictive labels. Models that assign future disease risk—even with high accuracy—can unintentionally shape identity, expectations, and healthcare access.
2. Regulatory Lag and Clinical Uncertainty—While the EU AI Act provides a framework for high-risk AI systems (7), it lacks pediatric-specific stipulations. Regulatory authorities must address critical issues such as the minimum age for data inclusion, dynamic consent models for growing children, and the long-term governance of digital twins.
3. The Changing Physician Role—As AI systems assume more diagnostic responsibility, clinicians may become safety overseers rather than decision-makers. This change challenges traditional models of accountability and requires rethinking medical education, trust building with families, and shared decision-making paradigms.

## 2 Discussion: A pediatric AI research and policy agenda

To ensure AI benefits children while protecting their rights, the pediatric research community should take the following steps:

1. Build a Pediatric AI Ethics Framework—Establish normative guidance on age-appropriate consent, the use of predictive modeling, and acceptable risk-benefit ratios for AI in minors. Input from bioethicists, legal scholars, and patient advocates is essential.
2. Advance Pediatric-Specific AI Development—Fund and support the creation of AI systems trained on pediatric data across age groups and conditions. Avoid extrapolating adult-trained models, which may introduce bias and misdiagnosis.
3. Implement Transparent, Inclusive Data Infrastructure—Create federated, secure, and diverse pediatric datasets with representative inclusion of vulnerable populations. Ensure data governance includes patient and parent stakeholders.
4. Reimagine Medical Education—Train pediatricians in AI fundamentals, algorithmic bias, and interpretability. Equip them to navigate co-decision-making with AI systems and to serve as ethical stewards.
5. Delay Broad Deployment of Digital Twins in Pediatrics—Until sufficient evidence and regulatory mechanisms are in place, digital twins should be confined to research or controlled pilot settings. Ethical impact assessments must precede scaling.

## 3 Conclusion

Artificial intelligence holds transformative potential for pediatric cardiology—but with it comes a distinct ethical imperative. The pediatric community must lead the development of age-sensitive standards for AI deployment. This moment offers a rare opportunity to shape a future in which children benefit from technological progress without compromising privacy, fairness, or trust.
